# The selective cyclooxygenase-2 inhibitor NS398 ameliorates cisplatin-induced impairments in mitochondrial and cognitive function

**DOI:** 10.3389/fnmol.2023.1295991

**Published:** 2023-11-29

**Authors:** Mohammad Abdur Rashid, Jason J. Tang, Ki-Hyun Yoo, Ana Corujo-Ramirez, Alfredo Oliveros, Sang Hoon Kim, Faheem Ullah, Raad Altawell, John R. Hawse, Peter D. Cole, Mi-Hyeon Jang

**Affiliations:** ^1^Department of Neurosurgery, Robert Wood Johnson Medical School, Rutgers, The State University of New Jersey, Piscataway, NJ, United States; ^2^Department of Neurologic Surgery, Mayo Clinic, Rochester, MN, United States; ^3^Department of Biochemistry and Molecular Biology, Mayo Clinic, Rochester, MN, United States; ^4^Division of Pediatric Hematology/Oncology, Rutgers Cancer Institute of New Jersey, New Brunswick, NJ, United States

**Keywords:** chemobrain, cisplatin, cyclooxygenase-2, NS398, mitochondria, cognitive impairment

## Abstract

Chemobrain is a condition that negatively affects cognition in cancer patients undergoing active chemotherapy, as well as following chemotherapy cessation. Chemobrain is also known as chemotherapy-induced cognitive impairment (CICI) and has emerged as a significant medical contingency. There is no therapy to ameliorate this condition, hence identification of novel therapeutic strategies to prevent CICI is of great interest to cancer survivors. Utilizing the platinum-based chemotherapy cisplatin in an investigative approach for CICI, we identified increased expression of cyclooxygenase-2 (COX-2) and prostaglandin E_2_ (PGE_2_) in the adult mouse hippocampus, and in human cortical neuron cultures derived from induced pluripotent stem cells (iPSCs). Notably, administration of NS398, a selective COX-2 inhibitor, prevented CICI *in vivo* without negatively affecting the antitumor efficacy of cisplatin or potentiating tumor growth. Given that dysfunctional mitochondrial bioenergetics plays a prominent role in CICI, we explored the effects of NS398 in cisplatin-induced defects in human cortical mitochondria. We found that cisplatin significantly reduces mitochondrial membrane potential (MMP), increases matrix swelling, causes loss of cristae membrane integrity, impairs ATP production, as well as decreases cell viability and dendrite outgrowth. Pretreatment with NS398 in human cortical neurons attenuated mitochondrial dysfunction caused by cisplatin, while improving cell survival and neurite morphogenesis. These results suggest that aberrant COX-2 inflammatory pathways may contribute in cisplatin-induced mitochondrial damage and cognitive impairments. Therefore, COX-2 signaling may represent a viable therapeutic approach to improve the quality of life for cancer survivors experiencing CICI.

## Introduction

Decades of intense biomedical investigation of cancer chemotherapy have improved survival for millions of cancer patients. Up to 75% of patients undergoing chemotherapy report cognitive impairment from active chemotherapy treatment. Consequently, improved cancer survival rates have revealed dysfunctions in learning, memory, and mood. This significant problem, known as or chemobrain or chemotherapy induced cognitive impairment (CICI), has been reported in up to 60% of cancer survivors (Argyriou et al., 2011; Horowitz et al., 2018). However, the underlying pathophysiological mechanism mediating CICI is poorly understood, and consequently, there is no known treatment to attenuate the symptoms of CICI.

In our previous screening of differential gene expression in adult mouse hippocampus upon cisplatin administration (Oliveros et al., 2022), we found that the *cyclooxygenase-2* gene was significantly up-regulated by cisplatin. Cyclooxygenase (COX), also known as prostaglandin-endoperoxide synthase (PTGS), generates prostaglandin and thromboxane from arachidonic acid via its two isoforms, COX-1 and COX-2. In most tissues, induction of COX-2 is activated upon pro-inflammatory signaling or injury, and implicated in neurodegeneration and cancer. In neurons, constitutive expression of COX-2 is integral to physiological function, as high basal levels of neuronal COX-2 in the hippocampus and cerebral cortex are reported to regulate synaptic plasticity and cognition (Yamagata et al., 1993; Breder et al., 1995; Kaufmann et al., 1996). Pathologically, neuronal COX-2 expression is potentiated by amyloid-β, glutamate, proinflammatory cytokines (Tocco et al., 1997; Pasinetti and Aisen, 1998; Bazan, 2001; Woodling et al., 2016). Moreover, prostaglandin E_2_ (PGE_2_), which is a major downstream product of COX-1 and COX-2, is reportedly increased in Alzheimer’s disease, thus demonstrating the critical nature of this pathway in neurodegeneration and aging-related cognition (Montine et al., 1999). Importantly, epidemiological and clinical data suggest that COX-2 inhibitors reduce Alzheimer’s disease risk, and other age-related diseases, notably cancer (Ulrich et al., 2006; Vlad et al., 2008). Preclinically, COX-2 inhibitors significantly improve synaptic and cognitive dysfunction in Alzheimer’s disease mouse models (Kotilinek et al., 2008), and marijuana-induced addiction (Chen et al., 2013). In contrast, the selective COX-2 inhibitor celecoxib is shown to have beneficial effects against age-related memory decline in clinical trials (Small et al., 2008). These observations suggest that COX-2 elevations contribute to neurodegenerative associated synaptic and cognitive impairments and given the resemblance between increased COX-2 expression in our CICI mouse model and neurodegenerative conditions, we explored whether COX-2 inhibition could have therapeutic benefits in CICI.

## Methods

### Mouse husbandry

In reflection that CICI is routinely reported by breast and ovarian cancer survivors, all experiments were performed on 3–4-month old female C57BL/6J mice (Jackson Laboratory) housed in standard, climate controlled ventilated mouse cages under a 12-h light/dark cycle, and provided water and food *ad libitum*.

### Xenograft tumor model

To test the *in vivo* effects of NS398 (10 mg/kg/day *i.p.*) and cisplatin (2.3 mg/kg/day *i.p.*) on tumor growth, MDA-MB-231 cells derived from a triple-negative breast cancer patient were obtained from American Type Culture Collection (ATCC) and orthotopically injected into the mammary fat pads of 4–5 month old CB17/Icr-Prkdc SCID female mice (Charles River, Strain Code: 236) at a concentration of ∼1 × 10^6^ cells in 100 μL + 100 μL of matrigel (Corning, Cat# 356231). Once tumor volumes reached approximately 50 mm^3^, mice were treated with 3 cycles of vehicle, cisplatin, cisplatin in combination with NS398 (CIS + NS398) or NS398 alone (VEH + NS398), as depicted. One day after drug regimen completion, tumors were isolated, and their weights were measured for tumor growth comparisons.

### Drugs

NS398 (Selective COX-2 inhibitor, 10 mg/kg *i.p*., Tocris Bioscience, Bristol, UK), was prepared in 15% DMSO (MilliporeSigma, St. Louis MO, USA), 15% Cremophor (MilliporeSigma) and 0.9% saline (MilliporeSigma). Cisplatin (Fresenius Kabi, Lake Zurich, IL, USA) was dissolved in 0.9% saline and all compounds were delivered at a 0.1 ml/10 g body weight.

### Cisplatin, methotrexate and NS398 administration

**FIGURE 1:**
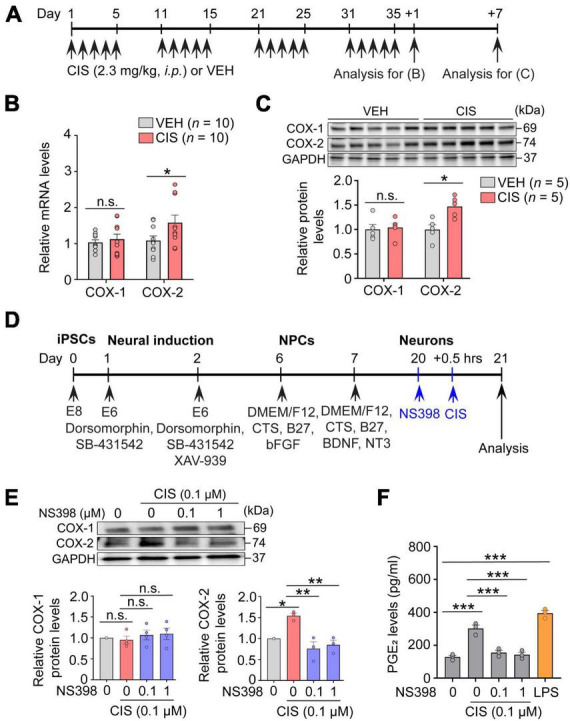
Cisplatin elevates COX-2 expression. **(A)** Experimental timeline showing cisplatin (CIS) or NS398 administration. **(B)** mRNA expression of COX-1 and COX-2 by qRT-PCR (*n* = 10 mice/group). **(C)** Western blot and densitometry for COX-1 and COX-2 (*n* = 5 mice/group). **(D)** Schematic showing differentiation procedures for cortical neurons derived from human induced pluripotent stem cells (hiPSC). **(E)** NS398 attenuates cisplatin-induced elevation of COX-2 protein in human excitatory cortical neurons. (*n* = 6 wells/group). **(F)** NS398 significantly blocked cisplatin-induced PGE_2_ production in human cortical neurons. Lipopolysaccharide (LPS: 10 μg/ml) was used as a positive control (*n* = 3 wells/group). Data represent mean ± SEM. Two-way ANOVA, Bonferroni’s **(B)** and Tukey’s **(C)** *post hoc c*orrection. One-way ANOVA, Tukey’s *post hoc c*orrection **(E,F)**. *P < 0.05, **P < 0.01, ***P < 0.001, n.s.: not significant. Circles in bar graphs represent western blot densitometry of an individual mouse brain **(B,C)**, or cell homogenates from individual culture wells **(E,F)**.

CICI is regularly experienced and reported in females undergoing and following breast and ovarian cancer (Falleti et al., 2005; Kesler and Blayney, 2016). Therefore, we focused on females for this study by employing the first-generation platinum-based compound cisplatin (Fresenius Kabi, Cat# 100351). Cisplatin has been a treatment for ovarian, testicular, lung, breast, and bladder cancer. Female mice (3–4 months of age) were administered 5 consecutive daily injections of cisplatin (2.3 mg/kg/day*, i.p*.) or vehicle, followed by 5 days without injections (i.e., 1 cycle of treatment). Mice were administered 3-to-4 cycles as appropriate for each experimental objective (Figure 1A). This cisplatin regimen comparably reflects clinical cancer treatment for this drug (Cavaletti et al., 1992; Szturz et al., 2019), making it a standard experimental regimen for rodent models of CICI (Oliveros et al., 2022) and neuropathic pain (Ta et al., 2009). Specifically, this dose regimen has been shown to possess antitumor efficacy, although significant dorsal root ganglion and hippocampal neuronal toxicity resulting in peripheral neuropathy and cognitive impairments has been reported (Zhou et al., 2016). NS398 (10 mg/kg/day, *i.p*., TOCRIS, Cat# 0942) was given 4 h prior to cisplatin administration for 3 cycles treatment regimen (Figure 1A).

For methotrexate (20 mg/kg/day*, i.p*.) administration (Supplementary Figure 1B), we modified a treatment protocol by Gibson et al. (2019), where we consecutively treated adult female C57BL/6J mice (3–4 months old) with methotrexate or vehicle for 5 days followed by 5 days without injections for 1-cycle (total cumulative dose of 100 mg/kg). We also treated a separate cohort of female mice for 3 cycles (total cumulative dose of 300 mg/kg) to ascertain whether early methotrexate accumulation (i.e., 1-cycle) could induce COX-2 expression changes.

### Quantitative RT-PCR and primer sequencing information

For quantitative RT-PCR analysis presented in Figure 1B, 3–4 month old female C57BL/6J mice were injected with cisplatin (2.3 mg/kg *i.p*.) or vehicle for 5 consecutive days followed by 5 days of rest from injections for 4 treatment cycles. Tissues were freshly collected (∼10–25 mg/tissue sample), snap frozen in dry ice, and stored at −80°C until processed. Total RNAs were extracted using Trizol reagent (Invitrogen, 221706) according to the manufacturer’s instructions. RNase-free DNase I (Thermo Fisher, EN0525)-treated total RNAs were used in cDNA synthesis using a SuperScript III First-strand Synthesis System (Invitrogen, 18080-051). Reverse transcription was carried out according to the manufacturer’s instructions in 20 μL reaction mixtures containing oligo(dT) primer. Quantitative real-time PCR was performed using Fast SYBR Green Master Mix (Applied Biosystems, 4385612) and the QuantStudio 3 Real-Time PCR sequence detection system (Thermo Fisher, A28136). The reaction was carried out in a total volume of 12 μL, which contained 6 μL of 2 × SYBR premix, 0.5 μL of each oligonucleotide primer and 1 μL of cDNA. The amplification conditions were an initial denaturation at 95°C for 10 min, followed by 40 cycles of 95°C for 15 s and 60°C for 60 s. The sequences of the sense and antisense primers for COX-1 and COX-2 were as follows:

***COX-1***

Forward: 5′-GAA TGC CAC CTT CAT CCG AGA AG-3′

Reverse: 5′-GCT CAC ATT GGA GAA GGA CTC C-3′

***COX-2***

Forward: 5′-GCG ACA TAC TCA AGC AGG AGC A-3′

Reverse: 5′-GCT CAC ATT GGA GAA GGA CTC C-3′

***Actin***

Forward: 5′-TTCTACAATGAGCTGCGTGTG-3′;

Reverse: 5′-GGGGTGTTGAAGGTCTCAAA-3′.

Following thermocycling, gene expression was analyzed using QuantStudio 3 Real-Time PCR sequence detection software, normalizing mRNA with Actin.

### Western blot analysis

For western blot analysis presented in Figures 1C, E, adult mouse hippocampus was magnetically homogenized (Storm 24 Bullet Blender, Next Advance, Troy, NY, USA) with 0.5 mm ZrO2 beads at a setting of 4 for 2 min in Neuronal Protein Extraction Reagent (N-PER, Thermo Scientific, Cat# 87792) containing HaltTM Protease Inhibitor Cocktail (Thermo Scientific, Cat# 78440) followed by centrifugation at 14,000× *g* for 15 min at 4°C. The supernatant protein concentrations were measured by the BCA assay kit (Pierce, Cat# 23227). Proteins in cell lysates (20 μg protein) were separated by 4–15% SDS-PAGE and electroblotted onto a nitrocellulose membrane for 60 min at 100 V at 4°C, which were blocked with 5% BSA for 60 min in TBST. Membranes were incubated overnight at 4°C with rabbit anti-COX1 (1:1000, Abcam, Cat# ab109025), rabbit anti-COX2 (1:1000, Cell Signaling Technologies, Cat# 12282S), and rabbit anti-GAPDH (1:1000, Santa Cruz Biotechnology, Cat# sc-25778) antibodies followed by HRP-linked secondary antibodies. Membranes were then washed and visualized with enhanced chemiluminescence (GE Healthcare Life Science, RPN2232). Target protein expression was normalized to that of control GAPDH expression.

### PGE_2_ assays

The concentration of prostaglandin E_2_ (PGE_2_) in culture medium (Figure 1F) was measured by ELISA according to the manufacturer’s instructions (Invitrogen, Catalog# KHL1701). Briefly, PGE_2_ standards and test samples were added mixed with an alkaline phosphatase (AP) conjugated-PGE_2_ antibody, and incubated shaking for 2 h at room temperature. Samples were washed after incubation, the excess reagents were washed, and pNpp substrate was added and absorbance was analyzed at 405 nm using a microplate reader. The PGE_2_ levels were expressed as pg/ml.

### Measurement of intracellular ATP content

**FIGURE 2:**
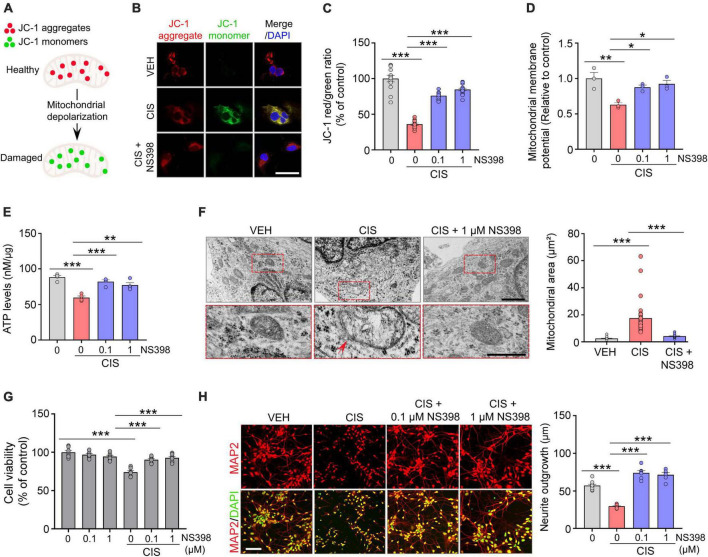
NS398 prevents cisplatin-induced mitochondrial dysfunction and impaired neuronal morphogenesis in human cortical neurons. **(A)** Illustration of reversible fluorescence of JC-1 aggregates (red, healthy cells) and monomers (green, damaged cells) indicative of mitochondrial membrane depolarization damage. **(B,C)** Representative images **(B)** and quantitation **(C)** show cisplatin decreased JC-1 aggregates, whereas NS398 increased JC-1 aggregates in human cortical neurons. Scale bar, 50 μm. (*n* = 3 wells/treatment). **(D)** NS398 restores cisplatin-induced deficits in mitochondrial membrane potential (*n* = 3 wells/treatment). **(E)** NS398 restores cisplatin-induced deficits in ATP levels (*n* = 4 wells/treatment). **(F)** Transmission electron microscopy identified enlarged mitochondria with severely disrupted cristae in cells treated with cisplatin (red arrow). Red dashed insets show a magnified view of cisplatin-induced mitochondrial enlargement and cristae disruption, which was prevented by NS398. All scale bars: 2 μm. **(G)** NS398 is neuroprotective against cisplatin-induced reduction in cell viability (*n* = 7 wells/treatment). **(H)** Representative confocal micrographs and quantitation shows NS398 prevents cisplatin-induced impairments in MAP2^+^ (red) neurite outgrowth in cortical neurons. DAPI (nuclei marker, green). Scale bar: 50 μm (*n* = 3 wells/treatment). Data represent mean ± SEM. One-way ANOVA, Tukey’s *post hoc* corrections. *P < 0.05, **P < 0.01, ***P < 0.001, n.s., not significant.

To assess intracellular ATP from iPSC derived human cortical neurons (Figure 2E), 1.0 × 10^5^ neurons were plated in 1.5 mL of culture medium, unless otherwise specified. On day 20, cortical cultures were treated with NS398 (0, 0.1, and 1 μM) for 30 min with or without pretreatment with cisplatin (0.1 μM) for 24 h. Subsequently, cells were lysed in buffer containing protease inhibitor cocktail (Cell Signaling) on ice for 5 min followed by centrifugation at 10,000 rpm for 5 min at 4°C. The supernatant protein concentrations were measured using the BCA assay kit (Pierce, Cat# 23227). Equal amounts of cell protein lysate from all treatments were loaded and ATP concentration was quantified in a 96-well plate using ATP bioluminescence according to the manufacturer’s instructions (Promega Cat # G9241). Lysates were incubated with a protease inhibitor cocktail (Cell Signaling) and CellTiter-Glo^®^ 2.0 reagent equal to the volume of cell supernatant, mixed for 2 min on an orbital shaker. To stabilize the luminescent signal, lysates were allowed to incubate at room temperature for 10 min. Intracellular ATP concentrations (nMol of ATP per μg protein) were measured via a luminometer (integration time of 0.25–1 s/per well). The value of the blank ATP sample wells was subtracted from each experimental sample.

### Statistical analysis

For statistical analysis of our results we utilized GraphPad Prism 9. For our molecular, cellular and behavioral assessments, we employed one-way ANOVA, or two-way ANOVA with and without repeated measures, followed by Bonferroni’s or Tukey’s *post hoc* multiple comparison testing, as appropriate for each experiment. Statistical significance was *P* ≤ 0.05 (*P ≤ 0.05, **P ≤ 0.01, ***P ≤ 0.001), while *P* > 0.05 is defined as not statistically significant (n.s.). All experiments and data analyses were performed in a blind fashion.

Additional methods are provided in Supplementary material.

## Results

### COX-2 expression levels were increased by cisplatin and methotrexate in the hippocampus and human excitatory cortical neurons

The essential function possessed by the hippocampus, among many others, is to act as an initial gatekeeper toward regulatory control of learning, memory and functional emotional behavior (Anacker and Hen, 2017). Several reports have also demonstrated it is particularly susceptible to chemotherapy-related neurotoxicity (Kesler et al., 2013). We therefore first examined alterations in COX-2 expression by cisplatin in freshly dissected adult hippocampus from mice that were administered cisplatin or vehicle (Figure 1A). In comparison to vehicle, qRT-PCR assessment showed that cisplatin significantly increased *COX-2* gene expression, while *COX-1* levels were unchanged (Figure 1B). Notably, western blot analysis also showed a selective increase in COX-2 protein levels by cisplatin at 7 days after cessation of cisplatin treatment, suggesting prolonged COX-2 induction following cisplatin administration (Figure 1C). To confirm neuronal COX-2 induction by cisplatin, human derived iPSC excitatory cortical neurons (Figure 1D) revealed that cisplatin significantly increases neuronal COX-2 in conjunction with increased PGE_2_ levels. COX-1 mRNA expression was unchanged upon cisplatin exposure. In contrast, cisplatin’s effect on COX-2 and PGE_2_ were significantly abolished by the potent and selective COX-2 inhibitor NS398 (Figures 1E, F). We next investigated whether COX-2 elevations were uniquely induced by the platinum-based chemotherapy cisplatin, or alternatively, if increased COX-2 expression was a general effect of chemotherapy induced neurotoxicity, regardless of the mechanism of action inherent to an individual class of chemotherapy. We found that similar to cisplatin, methotrexate, an anti-folate antimetabolite chemotherapy commonly used for acute lymphocytic leukemia and breast cancer, also significantly increases the expression levels of COX-2 *in vivo* and *in vitro* (Supplementary Figure 1). Since cisplatin and methotrexate are reported to cause cognitive impairments, and despite the different mechanisms of action engaged for cancer eradication by cisplatin (DNA-adduct formation) and methotrexate (antifolate antimetabolism), our data suggest that pathological activation of COX-2 signaling may be a common pathway of chemotherapy-induced neurotoxicity.

### NS398 prevents cisplatin-induced behavioral impairments

**FIGURE 3:**
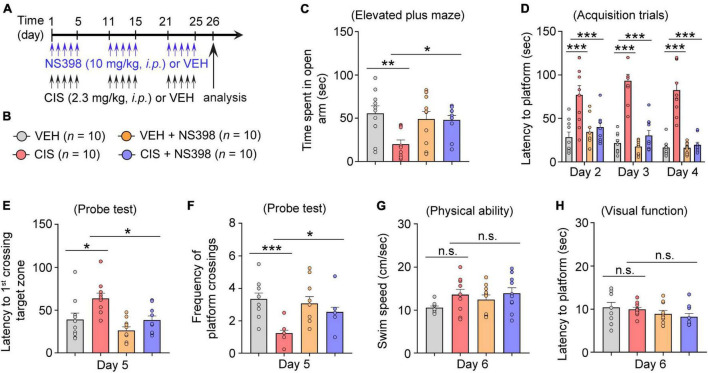
COX-2 inhibition through NS398 administration prevents cisplatin-induced anxiety and cognitive dysfunction. **(A,B)** Experimental timeline and groups (*n* = 10 mice/group). **(C)** Time spent in the open-arm of the elevated plus maze indicates NS398 prevents cisplatin-induced anxiety-like behavior. **(D)** NS398 prevents cisplatin-induced deficits in latencies to find the hidden platform during the Morris water maze (MWM; Day 2–4) spatial learning and memory acquisition. **(E,F)**, MWM probe test for memory recall (Day 5). **(G,H)** MWM swim-speed and latency to reach the platform (day 6) was similar between groups, confirming there were no physical or visual dysfunction. All values represent mean ± SEM. One-way ANOVA, Tukey’s *post hoc* correction. *P < 0.05, **P < 0.01, ***P < 0.001, n.s., not significant.

If COX-2 induction is a key mediator of cisplatin-induced impairments in behavioral function, we postulate that inhibiting COX-2 levels should prevent cisplatin-induced cognitive impairment. To test this hypothesis, adult female mice were pre-administered NS398 followed by cisplatin or vehicle for 3 cycles of treatment (Figure 3A). Anxiety-like behavior examination in the elevated plus maze (EPM) revealed that cisplatin caused decreased open arm time, while NS398 pretreatment increased open-arm time in cisplatin-treated mice (Figures 3B, C). Given that cisplatin-treated mice also exhibit reduced entries in both open and closed arms, suggestive of cisplatin-induced hypoactivity, we also analyzed percentage of time spent in the open arm to rule out potential confounding effects that may arise from hypoactivity. As shown in Supplementary Figure 2A, cisplatin-treated mice showed less percentage of time spent in open arm, suggesting that these mice had increased anxiety-like behavior in the EPM. Notably, NS398 prevents elevated anxiety-like behavior caused by cisplatin.

Next, using the Morris water maze (MWM), we evaluated functional spatial learning and memory ability. In this assay, cisplatin-treated mice exhibited longer times (latencies) to find the submerged escape platform (acquisition training Days 2–4). Hence, the increases in swimming time suggest that cisplatin-treated mice took longer to learn to find the escape platform, indicative of impaired learning. Conversely, pre-administered NS398 significantly ameliorated cisplatin-induced learning difficulties, as these mice exhibited shorter swim latencies to find the submerged escape platform (Day 2–4; Figure 3D). During the memory recall probe test (Day 5), in comparison to the vehicle alone, cisplatin-treated mice had significantly longer swim times to find and swim through the target zone (i.e., where the escape platform was previously located during training Days 2–4). However, pre-administered NS398 in cisplatin-treated mice significantly shortened swim times to reach the target zone (Figure 3E). In addition, during the memory recall probe test, we also found that cisplatin treatment significantly lowered the frequency of platform crossings in comparison to vehicle-alone, while NS398 pretreatment significantly reversed this behavior, thereby increasing the number of crossings, suggesting that NS398 improved memory recall (Figure 3F). To ascertain that our results are not caused by off-target drug effects from cisplatin or NS398, we examined swim speed and visual function across the treatment groups and did not detect differences in these measures (Figures 3G, H). This suggests that the data presented in this report confirms that NS398 or cisplatin specifically affect learning and memory function, as opposed to physical or visual capacity. Therefore, these results suggest a preventative role for NS398 against cisplatin-induced detriments to anxiety and spatial memory in adult female mice.

### Neuroprotective effects of NS398 in cisplatin-potentiated mitochondrial dysfunction, human cortical cell viability, and neurite morphogenesis *in vitro*

Mitochondrial dysfunction has a prominent role in chemotherapy-related neurotoxicity (Rashid et al., 2022). Because mitochondrial membrane potential (MMP) is a key indicator of mitochondrial health and function, we analyzed the effects of cisplatin and NS398 in human cortical neurons using the cationic fluorescence marker for MMP, tetraethylbenzimidazolylcarbocyanine iodide (JC-1). Our confocal imaging analysis showed that in vehicle treated cortical neurons which possess intact membrane potential, JC-1 forms aggregated red fluorescence in healthy mitochondria (Figures 2A, B). However, cisplatin treated cortical neurons exhibit significant impairments in MMP, as evidenced by JC-1 expression transitioning from red-fluorescent aggregates to green-fluorescent monomers, which is indicative of cisplatin-induced neurotoxicity on mitochondria. The change in ratio of red to green fluorescence is used as an indicator of mitochondrial condition. Notably, while treatment of cisplatin to human cortical neurons resulted in increased green fluorescence (JC-1 monomers) intensity and reduced red fluorescence (JC-1 aggregates), pre-treatment with NS398 reversed this phenotype, as our quantitation found increased red fluorescence and reduced green fluorescence, indicating that NS398 protected against the mitochondrial membrane depolarization induced by cisplatin (Figure 2C). Assessment of MMP also demonstrated that cisplatin significantly decreased MMP in human cortical neuronal cultures, while in contrast, NS398 prevented membrane potential disruption (Figure 2D). Therefore, our reported data emphasizes how NS398 may be efficacious in inhibiting cisplatin-caused disruptions to MMP in human cortical neurons.

Consistent maintenance of MMP is an essential process for homeostatic release of ATP. Disruptions to homeostatic ATP production is associated with neuropathologies in Alzheimer’s disease and Parkinson disease (Zhao et al., 2019; Misrani et al., 2021). In agreement with disruptions to ATP homeostasis in neurodegenerative conditions, we detected significantly reduced levels of ATP in human cortical neuronal cultures exposed to cisplatin. In contrast, NS398 prevented these adverse effects on ATP generation (Figure 2E), indicating that NS398 attenuates cisplatin-potentiated disruptions to ATP homeostasis in human cortical neurons.

Mitochondrial swelling is a markedly prominent ultrastructural change following neural injuries, and this phenotype is considered a hallmark of mitochondrial dysfunction (Zhao et al., 2019). To investigate the effect of cisplatin and NS398 on mitochondrial ultrastructural changes in human cortical neurons, we employed transmission electron microscopy (TEM). TEM indicated a significant expansion of mitochondrial space lacking a well-defined regular matrix structure caused by cisplatin, although these ultrastructural defects were abolished by NS398 pretreatment (Figure 2F). Therefore, it is noteworthy that our data implies NS398 effectively preserves mitochondrial structure, function, and morphology in spite of changes caused by cisplatin exposure.

To determine the neuroprotective properties of NS398 on chemotherapy-induced neurotoxicity, we analyzed cell viability and morphological examination of neurite outgrowth. As depicted in Figure 2G and Supplementary Figure 3, human cortical neuronal cultures exposed to cisplatin exhibited significant reduction in cell viability while importantly, NS398 alone did not yield this effect. Moreover, pre-treatment with NS398 was efficacious in attenuating cisplatin-induced suppression of cell survival. In addition, we assessed neurite length in our cortical human neuronal cultures and found that while cisplatin impaired neurite outgrowth, pre-treatment with NS398 significantly prevented cisplatin-induced neurite outgrowth impairment (Figure 2H). Taken together, the data presented here emphasizes how in human cortical neurons, NS398 promotes neuroprotection against cisplatin-induced cell viability defects and neuronal morphogenesis.

### No impact of NS398 on tumor growth or anti-tumor activity of cisplatin

**FIGURE 4:**
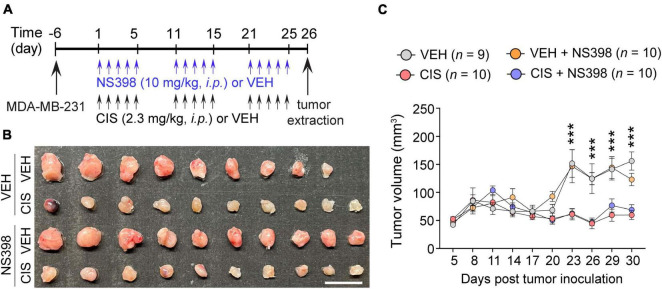
No impact of NS398 on tumor growth or anti-tumor efficacy of cisplatin. **(A)** Experimental timeline schematic. **(B)** MDA-MB-231 xenograft tumor treatment comparison from SCID mice (Scale bar: 1 cm.). **(C)** Tumor volume measurement (*n* = 9–10 mice/group). Results reported in mean ± SEM. Two-way ANOVA, Tukey’s *post hoc* corrections. ***P < 0.001 (vehicle vs. cisplatin).

To ascertain that NS398 did not induce aberrant tumor expansion or inhibit cisplatin’s antitumor efficacy, adult severe combined immunodeficiency (SCID) female mice were implanted with triple-negative (MDA-MB-23) breast cancer cell lines. We then administered NS398 and cisplatin (Figure 4A). As expected, cisplatin inhibited tumor growth, and importantly, NS398 treatment in either vehicle-treated mice or cisplatin-treated mice did not significantly alter tumor size (Figures 4B, C). The data from these experiments imply that NS398 neither promotes tumor growth nor interrupts cisplatin’s antitumor properties, allowing us to conclude that NS398 possesses is adequately safe to use within the dose range we utilized for our cognitive behavior testing.

## Discussion

In this report, we provide the first evidence demonstrating a causative role for COX-2-mediated PGE_2_ signaling in cisplatin-induced cognitive deficits, and a therapeutic potential for NS398-mediated COX-2 inhibition in preventing cisplatin-induced cognitive impairment. There are several important implications from our study.

First, given that COX-2-mediated PGE_2_ inflammatory signaling is widely implicated in neurodegeneration, our study expands a new causative role for COX-2 and downstream PGE_2_ inflammatory signaling in CICI. In support of our findings, selective inhibition of COX-2 by celecoxib demonstrated benefits against aging-related memory decline in preclinical studies and clinical trials (Small et al., 2008). In agreement with these findings, we show that cisplatin significantly upregulates COX-2 expression and PGE_2_ levels in the adult mouse hippocampus and human derived cortical neuronal cultures. Consequently, in the present study, cisplatin-treated mice show increased anxiety and impaired spatial memory, phenotypes that are consistent with our reported observations in our prior reports (Oliveros et al., 2022). Remarkably, the selective COX-2 inhibitor NS398 significantly prevents cisplatin-induced behavioral impairments, suggesting that neuronal COX-2 induction may have a key role in chemobrain, making inhibition of COX-2 a viable therapeutic approach.

Second, our findings identify that cisplatin-impaired mitochondrial bioenergetics and neuroprotection by COX-2 inhibition is an underlying mechanism in chemobrain. Cisplatin activates neuronal DNA damage responses, produces mitochondrial ROS, lowers ATP generation, and induces loss of MMP (Zhao et al., 2019; Misrani et al., 2021; Rashid et al., 2022). In line with these observations, our current study showed that NS398 attenuated cisplatin-induced mitochondrial oxidative stress, loss of MMP, and reduced ATP generation. Therefore, NS398 exerts potent anti-inflammatory and antioxidant properties that protect against cisplatin-induced neurotoxicity.

Lastly, COX-2-mediated PGE_2_ signaling has been implicated in cancer development. A number of studies reveal that aberrantly high COX-2 expression is associated with excessive tumor growth and resistance of malignancies to chemotherapy and radiotherapy (Hashemi Goradel et al., 2019). Recent studies have found that PGE_2_ in the tumor microenvironment actively triggers tumor immune evasion, thus inhibition of COX-2 may enhance immunotherapy (Zelenay et al., 2015). Preclinically, COX-2 inhibitors show promise in enhancement of anti-tumor responses (Sharma et al., 2005). Moreover, COX-2 inhibitors are currently undergoing clinical trials for enhancing anti-tumor activity when combined with other chemotherapies or PD-L1 inhibitors. Importantly, our results show that NS398 neither promotes tumor growth, nor interrupts the anti-tumor activity of cisplatin. Therefore, it is of importance to determine whether chronic NS398 treatment can suppress tumor growth and/or synergistically enhance chemotherapy’s anti-tumor activities. If this is the case, COX-2 inhibition through NS398 administration may be a multi-pronged therapeutic strategy for preventing both CICI and cancer.

## Data availability statement

The raw data supporting the conclusions of this article will be made available by the authors, without undue reservation.

## Ethics statement

Ethical approval was not required for the studies on humans in accordance with the local legislation and institutional requirements because only commercially available established cell lines were used. The animal study was approved by Lauren Zizza, Rutgers University. The study was conducted in accordance with the local legislation and institutional requirements.

## Author contributions

MR: Conceptualization, Data curation, Formal analysis, Investigation, Methodology, Supervision, Validation, Visualization, Writing – original draft, Writing – review and editing. JT: Data curation, Formal analysis, Investigation, Methodology, Validation, Writing – review and editing. K-HY: Data curation, Formal analysis, Investigation, Methodology, Validation, Writing – review and editing. AC-R: Formal analysis, Investigation, Validation, Writing – review and editing. AO: Data curation, Investigation, Methodology, Software, Validation, Writing – review and editing. SK: Formal analysis, Methodology, Supervision, Validation, Writing – review and editing. FU: Supervision, Validation, Writing – review and editing. RA: Formal analysis, Validation, Writing – review and editing. JH: Conceptualization, Data curation, Formal analysis, Investigation, Methodology, Supervision, Validation, Writing – review and editing. PC: Conceptualization, Funding acquisition, Supervision, Validation, Writing – review and editing. M-HJ: Validation, Visualization, Writing – original draft, Writing – review and editing, Conceptualization, Data curation, Funding acquisition, Resources, Supervision.
