# Supplementary material for: The selective cyclooxygenase-2 inhibitor NS398 ameliorates cisplatin-induced impairments in mitochondrial and cognitive function
Source: Front Mol Neurosci. 2023 Nov 29;16:1295991. doi: 10.3389/fnmol.2023.1295991 (PMC10716194; doi:10.3389/fnmol.2023.1295991)
Supplement: Supplementary file 1 [file Data_Sheet_1.PDF]

Supplementary Information

The selective cyclooxygenase-2 inhibitor NS398 ameliorates cisplatin-induced impairments in mitochondrial and cognitive function

Mohammad Abdur Rashid[1,2†], Jason J. Tang[2†], Ki-Hyun Yoo[1,2], Ana Corujo-Ramirez[2], Alfredo Oliveros[1,2], Sang Hoon Kim[1], Faheem Ullah[1], Raad Altawell[1], John R. Hawse[3], Peter D. Cole[4], and Mi-Hyeon Jang[1,2,3,#].

[1]Department of Neurosurgery, Robert Wood Johnson Medical School, Rutgers, The State University of New Jersey, Piscataway, NJ, United States
[2]Department of Neurologic Surgery, Mayo Clinic, Rochester, MN, United States
[3]Department of Biochemistry and Molecular Biology, Mayo Clinic, Rochester, MN, United States
[4]Division of Pediatric Hematology/Oncology, Rutgers Cancer Institute of New Jersey, New Brunswick, NJ, United States

[†]These authors contributed equally to this work and share first authorship.

Supplemental material includes

- Additional materials and methods
- Supplementary Figure S1
- Supplementary Figure S2
- Supplementary Figure S3

Additional materials and methods

Differentiation of Induced Pluripotent Stem Cell (iPSC) into Cortical Neurons

Human iPSCs (004-BIOTR-0002) derived from a 16-year-old male without any detectible neurological diagnosis were obtained from the Biotrust at Mayo Clinic Center for Regenerative Medicine. As described in our previous methods (Yoo et al., 2021, Rashid et al., 2022), iPSCs were grown onto recombinant human vitronectin (rhVTN)-coated T-25 flasks in Essential 8™ Medium (ThermoFisher Scientific) and passaged routinely (at ~60% confluency) using hypertonic citrate buffer (4.4g/L sodium citrate, 24g/L potassium chloride; osmolality: 630-640 mOsmol/kg).

For neural induction, as shown in Figure 1D, iPSC cultures were passaged and plated onto rhVTN-coated 6 well plates in Essential 8™ medium. After 15-24 hours, the medium was exchanged for Essential 6™ medium supplemented with 2 μM dorsomorphin (Sigma-Aldrich, P5499) and 6 μM SB-431542 (Tocris, 1614) for 24 hours. 24 hours following neural induction, the medium was exchanged daily for Essential 6™ medium supplemented with 2 μM dorsomorphin (Sigma Aldrich, P5499), 6 μM SB-431542 (Tocris, 1614), and 2 μM XAV-939 (Tocris, 3748) for 4 days. On day 6, neural induction medium was replaced by DMEM/F12 (ThermoFisher Scientific, 110-39021) supplemented with 20 ng/ml fibroblast growth factor (FGF; R&D Systems, 233-FB), N2 (CTS, 100X; ThermoFisher Scientific, A13707-01) and B27 (serum free, 50X, ThermoFisher Scientific, 17504044) for 24 hrs.

To promote differentiation of the neural progenitors into cortical neurons, the neural medium was treated with 20 ng/ml brain-derived neurotrophic factor (BDNF; Peprotech, 450-02) and 20 ng/ml Neurotrophin-3 (NT3, Peprotech, 450-03) for 10 days (until day 18) with medium changes every 2 days. At Day 18, cultures were passaged with 0.5 mM EDTA (0.02% DPBS) and plated onto rhL521 (Life Science, 354222)-coated MatTek dishes/plates in DMEM/F12 supplemented

with N2 and B27 containing 20 ng/ml BDNF (Peprotech, 450-02) and 20 ng/ml NT3 (Peprotech, 450-03) for 2 days (until day 20).

Cell viability presented in Figure 3G was determined using CellTiter 96®AQueous One Solution Cell Proliferation Assay (Promega, Cat# G3582) according to the manufacturer's protocol. Vehicle-treated cells were set to 100% viability.

For immunofluorescence staining presented in Figure 3H, human excitatory cortical neurons were fixed with 4% paraformaldehyde in PBS (pH 7.4) for 30 min at 25°C. Cells were washed with 0.1M Tris-buffered saline (TBS, pH 7.5), and then blocked with 10% (vol/vol) normal goat serum in TBS containing 0.3% (vol/vol) Triton X-100 (60 min at 25°C). The fixed cells were incubated with anti-rabbit MAP2 primary antibody (1:300, EMD Millipore, Cat# AB5622) at 4°C overnight. Cells were subsequently washed with TBS and incubated with Alexa Fluor 488 or 555- conjugated secondary antibodies at 25°C for 60 min. To visualize nuclei, the cells were mounted with ProLong Gold antifade mounting medium with DAPI. Neurite outgrowth was evaluated by immunostaining coupled to confocal microscopy using the Zeiss LSM800 + AiryScan confocal laser microscope (Carl Zeiss), and average neurite lengths were quantified using Zeiss ZEN software.

Behavioral analysis

Mice were transferred from colony housing to the behavior testing suite and allowed acclimation to this suite for 1 hr before the onset of testing to minimize stress during behavior testing. All tests were performed during the light phase (6 am- to- 6 pm) of the 12-hr light/dark cycle. All behavior experimental data was video recorded with a monochrome camera mounted to the ceiling of the testing suite and analyzed with EthoVision-XT 14 video tracking (Noldus Information Technology, Leesburg, VA, USA). Where appropriate, testing chambers were

sanitized with 70% ethanol between trials. At the end of each behavior testing assay, mice were returned to their home cage. Following completion of daily behavior testing, mice were returned to colony housing.

Elevated plus maze (EPM): The elevated plus maze (ENV-560A, Med-Associates St. Albans VT, USA) presented in Figure 2C, takes advantage of the proclivity for rodents to avoid open, unprotected areas, making this simple and efficient test appropriate to measure anxiety-like behavior. The EPM is elevated 50 cm above the floor and consists of two open arms and two closed arms of similar dimensions (L 34.9 cm x W 6 cm). The closed arms are lined with a 26 cm high opaque black wall. Mice are released at a center location (L 6 cm x W 6 cm) between the open and closed arms and allowed to freely explore every section of the maze for 5 min. Time spent in the open and closed arms of the maze was video tracked and recorded (Noldus).

Morris Water Maze (MWM): MWM is a rodent behavioral procedure to study spatial learning and memory. The MWM has proven to be a robust and reliable test that is strongly correlated with hippocampal-dependent cognitive function (Clark et al., 2007, Vorhees and Williams, 2006). The arena setting is a round pool (120 cm diameter and 90 cm deep) filled with water mixed with non-fat dry milk powder to make opaque water. A video camera (Noldus) set up directly above the water maze ensured video tracking swim behavior in mice within the camera's field of view. To facilitate spatial memory function, visible spatial cues were placed at opposite coordinate points of the circular pool and designated as North (N), East (E), South (S), West (W), thus dividing the pool into four quadrants of equal dimensions, with each quadrant designated as Northeast (NE), Southeast (SE), Southwest (SW), Northwest (NW). Each coordinate point (N, E, S, W) and corresponding spatial cues was equidistant between NE, SE, SW, NW release

points. A submerged escape platform (30 cm high) was placed in the center of the SE quadrant. During each testing day, the water temperature was maintained between 25°C to 28°C. As shown in Figure 2D-H, the test was conducted in 6 consecutive days consisting of visible platform days (Day 1 and Day 6), training days (Days 2–4), and a memory probe day (Day 5). Each test day, mice would undergo 4 swimming trials per day for a duration of 2 min/trial, and interspaced with a 2–5 min interval between each trial, totaling 4 trials/day. Each trial commenced following releasing each test subject in a clockwise fashion, from NE, SE, SW, NW quadrant release points. Mice that failed to find the location of the visible or submerged (hidden) escape platform in each trial, were manually guided to the platform and allowed to remain there for 30 seconds to facilitate learning the location of the submerged platform. Briefly, on Day 1 for habituation, the escape platform was placed slightly above the water level with an orange flag pole to signal its location, thus mice were able to see and learn the platform location to escape the water. On Days 2–4, mice underwent acquisition training for spatial learning to find the hidden escape platform, which was set 1 inch below the water level, so mice were trained to rely on spatial cues to learn to find the submerged platform. The escape latency, which is the time spent finding the platform, was recorded for further data analysis. If mice failed to find the submerged platform in 2 min, their latency was recorded as 2 min and they were guided to the platform and allowed to stay there for 30 sec. On Day 5 for the memory probe test, the escape platform was removed to determine whether mice relied on spatial cues and learned to memorize the position of the hidden platform. The frequency of crossing the platform zone and latency to find the target platform zone was recorded for data analysis. On Day 6, we tested for visual acuity where the escape platform was set slightly above the water level and signaled by flag of its location. The latency to find this visible platform was recorded.

Determination of mitochondrial membrane potential

The mitochondrial membrane potential of human cortical neurons presented in Figure 3A-D was assessed with a 5,5,6,6'-tetrachloro-1,1',3,3' tetraethylbenzimi-dazoylcarbocyanine iodide (JC-1), a cationic dye that accumulates in energized mitochondria, that is widely used as a sensitive marker for MMP (Lee et al., 2020, Rashid et al., 2022), using mitochondrial membrane potential assay kit (Abcam, ab113850) according to the manufacturer's instructions. The cells were incubated with 10 μmol/L JC-1 dye for 20 minutes at 37°C and washed twice with 1X dilution buffer. Fluorescence was visualized using the Zeiss LSM800 + AiryScan confocal laser microscope (Carl Zeiss). The samples were assessed on a fluorescent plate reader (Molecular Device-Soft max) with a set excitation wavelength at 488/530 nm (green) for monomers and 525/590 nm for aggregates (red), and the membrane potential was represented as the ratio of red/green fluorescence intensity.

Transmission electron microscopy

For transmission electron microscopy presented in Figure 3F, human cortical neurons were plated onto a glass coverslip in a six-well plate. Cells were fixed in 4% paraformaldehyde + 1% glutaraldehyde in 0.1 M phosphate buffer, pH 7.2 for 12 hours at 4 °C. Further processing was performed with the aid of a PELCO BioWave® laboratory microwave oven (Ted Pella, Inc., Redding, CA) operating at 250W. Fixed cells were microwaved under vacuum in 1% osmium tetroxide in 0.1 M phosphate buffer for the sequence: 2 min on; 2 min off; 2 min on; 15 min off. Cells were rinsed in nH_2O and microwaved in 2% uranyl acetate using the previous sequence. Following another nH_2O rinse, cells were dehydrated using the graded ethanol series (70%, 80%, 95%, 100%, 100% acetone). For each dehydration step, cells were microwaved for 40 sec and allowed to rest at RT for 2 min. Cells were infiltrated with Embed 812/Araldite resin

(EMS, Hatfield, PA) by microwaving under vacuum 2 min on, 2 min off, 2 min on, 30 min off, in 2:1, 1:1 and 3:1 ratio of acetone:resin sequentially. After a final incubation in 100% resin for 12 hours at room temperature, cells were embedded by inverting resin filled embedding molds atop the monolayer and allowing the resin to polymerize for 24 hours at 60 °C. Ultrathin sections (0.1 micron) were stained with lead citrate for 5 min at room temperature. Micrographs were acquired using a JEOL 1400+ Transmission Electron Microscope (JEOL, Inc., Peabody, MA) at 80 kV equipped with a Gatan Orius camera (Gatan, Inc., Warrendale, PA).

Supplementary Figures and Legends

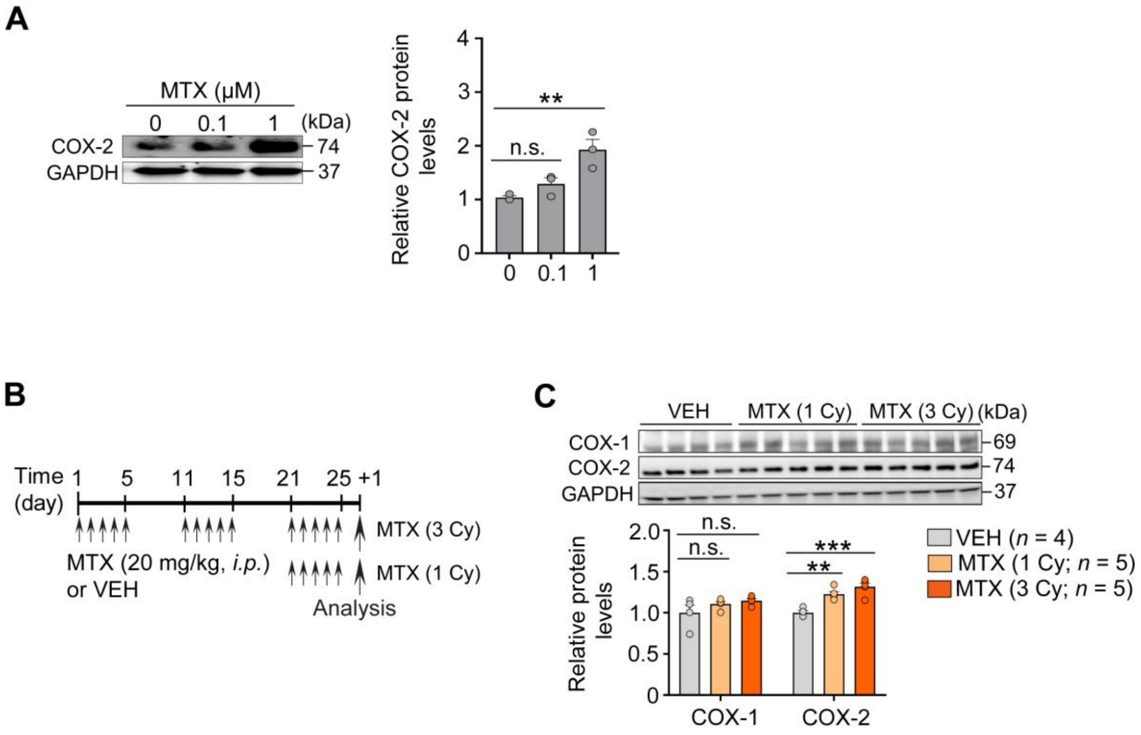

Supplementary Figure S1. Dose-dependent effect of methotrexate on expression of COX-2 *in vitro* and *in vivo*.
(A) Representative western blots and densitometry quantification in human excitatory cortical neurons derived from hiPSCs *in vitro*. Cells were treated with methotrexate (MTX) at a dose of 0.1 and 1 μM, respectively, and analyzed 24 hour later. *n = 3* wells/dose. **(B)** Schematic depicting the experimental timeline of methotrexate administration *in vivo*. Adult female C57BL/6J mice at 3-4 months old were intraperitoneally (*i.p.*) administered with vehicle or methotrexate (20 mg/kg *i.p.*) consisting of 5 daily injections for 1 cycle (1 Cy; for a total cumulative dose of 100 mg/kg) followed by 5 days without injections and repeated for 3 cycles (3 Cy; a total cumulative dose of 300 mg/kg). **(C)** Representative western blots and densitometry quantification derived from female adult mouse hippocampus treated with for either 1 cycle or 3 cycles of methotrexate or vehicle *in vivo*. In bar graphs from (A) *in vitro* cortical studies and (C) *in vivo* mouse studies, circles within each graph represent an individual well (A) or an individual brain (C), respectively. Data represent mean ± SEM. One-way ANOVA followed by Tukey's *post-hoc* correction (A) and two-way ANOVA (C) followed by Tukey's *post-hoc* correction. *: $P < 0.01$, **: $P < 0.01$, ***: $P < 0.001$, n.s.: not significant.

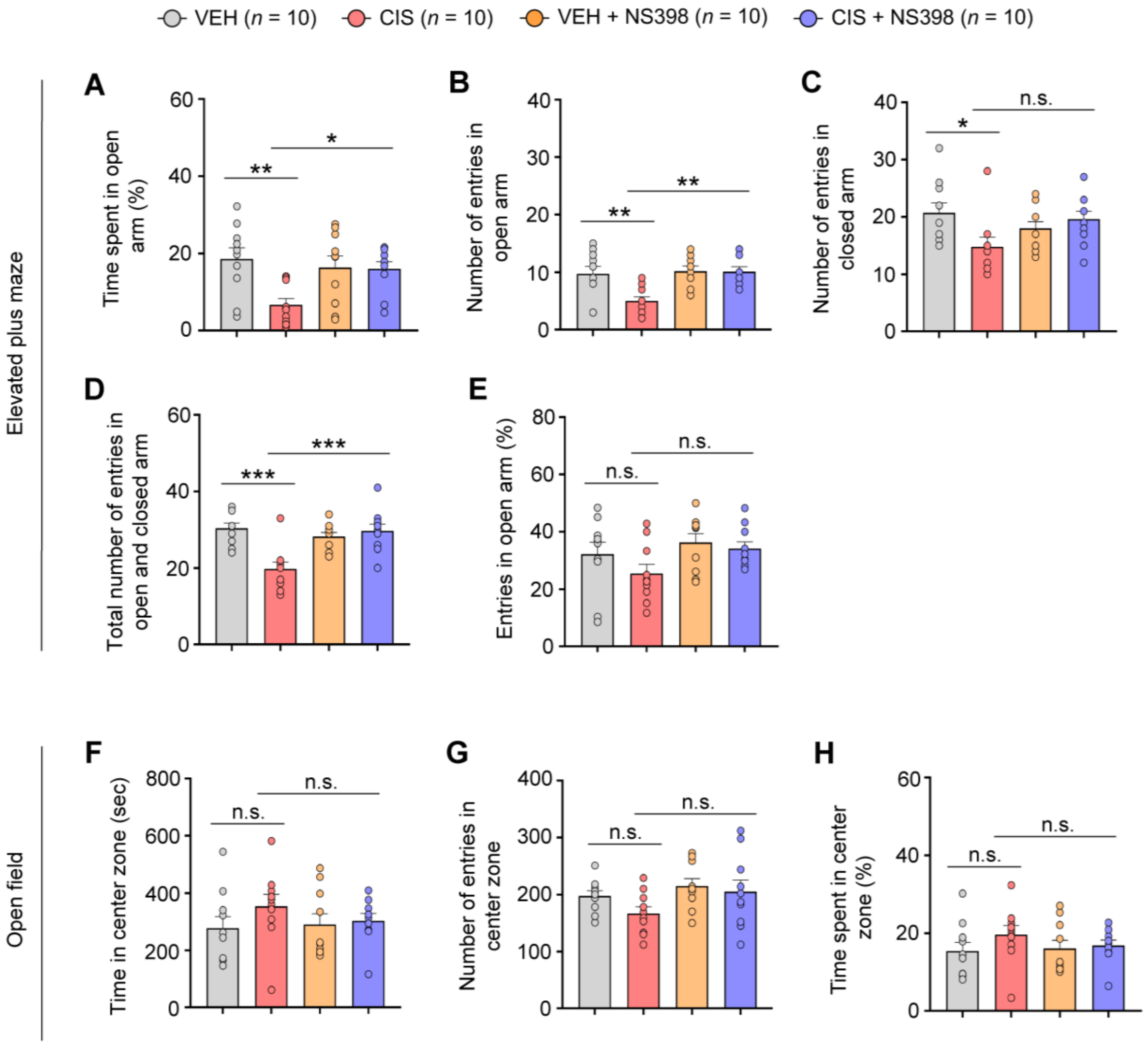

Supplementary Figure S2. Effects of NS398 on cisplatin-induced anxiety.
(A-E) Elevated plus maze. Percentage of time spent in the open arm (A), number of entries in the open arm (B), closed arm (C), and both open and closed arm (D) as well as percentage of entries in open arm (E). (F-H) Open field test. Time spent in center zone (F), number of entries in center zone (G) and percentage of time spent in center zone (H). All values represent mean ± SEM. One-way ANOVA, Dunnett's *post-hoc* correction. *: $P < 0.05$, **: $P < 0.01$, ***: $P < 0.001$, n.s.: not significant.

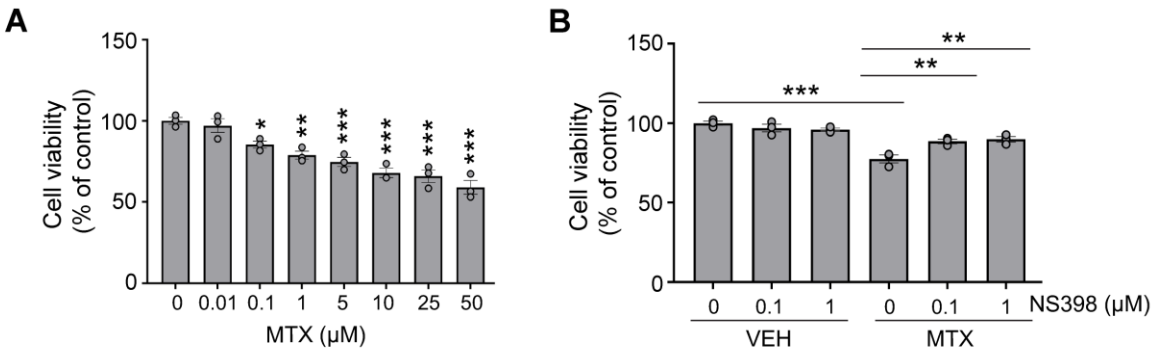

Supplementary Figure S3. Neuroprotective effects of NS398 against methotrexate-induced neurotoxicity *in vitro*.
(A) Dose-dependent neurotoxic effects of methotrexate on cell survival in human cortical neurons derived from induced pluripotent stem cells (iPSCs). n = 3 wells. **(B)** Neuroprotective effects of NS398 against methotrexate-induced reduction in cell survival. $n=3$ wells/dose. Data represent mean ± SEM. Circles within bar graphs represent an individual well. One-way ANOVA followed by Dunnett's (A) and Tukey's (B) *post-hoc* correction. *: $P < 0.05$, **: $P < 0.01$, ***: $P < 0.001$.

References

Clark, R. E., Broadbent, N. J. and Squire, L. R. (2007). The hippocampus and spatial memory: findings with a novel modification of the water maze. *J Neurosci,* 27**,** 6647-6654.

Lee, Y. J., Kim, G. H., Park, S. I. and Lim, J. H. (2020). Down-regulation of the mitochondrial i-AAA protease Yme1L induces muscle atrophy via FoxO3a and myostatin activation. *J Cell Mol Med,* 24**,** 899-909.

Rashid, M. A., Oliveros, A., Kim, Y. S. and Jang, M. H. (2022). Nicotinamide Mononucleotide Prevents Cisplatin-Induced Mitochondrial Defects in Cortical Neurons Derived from Human Induced Pluripotent Stem Cells. *Brain Plast,* 8**,** 143-152.

Vorhees, C. V. and Williams, M. T. (2006). Morris water maze: procedures for assessing spatial and related forms of learning and memory. *Nat Protoc,* 1**,** 848-858.

Yoo, K. H., Tang, J. J., Rashid, M. A., Cho, C. H., Corujo-Ramirez, A., Choi, J., et al. (2021). Nicotinamide Mononucleotide Prevents Cisplatin-Induced Cognitive Impairments. *Cancer Res,* 81**,** 3727-3737.
